# Idarubicin and cytarabine in combination with gemtuzumab ozogamicin (IAGO) for untreated patients with high-risk MDS or AML evolved from MDS: a phase II study from the EORTC and GIMEMA Leukemia Groups (protocol 06013)

**DOI:** 10.1007/s00277-015-2486-9

**Published:** 2015-09-26

**Authors:** Theo de Witte, Stefan Suciu, Liv Meert, Constantijn Halkes, Dominik Selleslag, Dominique Bron, Sergio Amadori, Roel Willemze, Petra Muus, Frédéric Baron

**Affiliations:** Radboud University Medical Centre, Nijmegen, The Netherlands; EORTC Headquarters, Brussels, Belgium; Leiden University Medical Center, Leiden, The Netherlands; St Jan’s Hospital, Brugge, Belgium; Institut J. Bordet (ULB), Brussels, Belgium; Tor Vergata University Hospital, Rome, Italy; University of Liege, Liege, Belgium; Department of Tumorimmunology, Radboud Institute of Molecular Life Sciences, Radboud University Medical Centre, PO Box 9101, 6500 HB Nijmegen, The Netherlands

**Keywords:** High-risk myelodysplastic syndromes, Secondary acute myeloid leukemia, Gemtuzumab ozogamicin, Chronic myelomonocytic leukemia, Liver toxicity, Cytogenetic risk score

## Abstract

The primary objective of this trial was to assess the feasibility, toxicity profile, and antitumor activity of gemtuzumab ozogamicin (GO) combined with a chemotherapy remission-induction regimen in adults with untreated high-risk myelodysplastic syndrome (HR-MDS) or secondary acute myeloid leukemia (sAML). In this phase II trial, 30 patients with median age of 58 years received 1 day of GO as a 1-h infusion at the dose level of 5 mg/m^2^ on day 7 of the remission-induction course further consisting of a continuous infusion of cytarabine 100 mg/m^2^/day for 10 days and idarubicin 12 mg/m^2^/day on days 1, 3, and 5. A consolidation course, consisting of intermediate-dose cytarabine (A) and idarubicin (I) followed by hematopoietic stem cell transplantation (HSCT) was planned for patients in complete remission (CR). The primary endpoints were response rate (CR/CRi) and severe toxicity rate. The secondary endpoint(s) were survival and progression-free survival (PFS) from start of treatment. Thirteen patients (43 %) achieved CR (eight patients) or CR with incomplete hematopoietic recovery (CRi) (five patients). In patients who achieved CR or CRi, the median time to recovery of neutrophils to 0.5 × 10^9^/l and of platelets to >50 × 10^9^/l was 29 and 30 days, respectively. Grade 3 to 4 severe toxicities occurred in nine patients. The most prominent was liver toxicity, as shown by elevated bilirubin levels in 16 patients and one case of nonfatal veno-occlusive disease (VOD). All 13 patients with CR/CRi received consolidation therapy, which was followed by allogeneic HSCT in five patients and autologous HSCT in three patients. According to the statistical design of the study, the idarubicin and cytarabine in combination with gemtuzumab ozogamicin (IAGO) regimen did not show sufficient activity to warrant further exploration of this regimen in adult patients with HR-MDS or sAML.

## Introduction

AML-like therapy for patients with high-risk myelodysplastic syndrome (MDS) has been accepted less widely than for patients with de novo AML [1, 2]. The European LeukemiaNet guidelines on MDS recommend that induction chemotherapy should be considered for fit patients without a suitable donor who are younger than age 65 to 70 years and have 10 % or more bone marrow blasts without adverse cytogenetic characteristics [3]. Prognostic factors for outcome after AML-like therapy in MDS have been studied less frequently than in de novo AML [2, 4–6]. Cytogenetic abnormalities, age, antecedent hematological disease (AHD), performance status, and applied treatment are prognostic factors for survival and event-free survival (EFS) [7]. The results of intensive chemotherapy in patients with advanced stages of MDS have improved with complete remission (CR) rates now ranging between 44 and 64 % [4, 7–11]. Remission after chemotherapy usually lasts less than 12 months [9, 12]. The higher incidence of adverse cytogenetic characteristics and the higher expression of the multidrug resistance 1 gene (MDR1) in MDS compared to de novo AML may explain the inferior response to chemotherapy [13–16].

The rationale for this study was to improve eradication of the malignant clones in high-risk MDS and secondary AML (sAML). Incomplete eradication of these clones is the main cause of treatment failure demonstrated as a relatively low CR rate and a high early relapse rate of more than 50 %, unless treated by allogeneic hematopoietic stem cell transplantation (HSCT). Therefore, new studies should focus on new and better remission-induction and consolidation regimens. For the development of a new prospective, randomized study, it is necessary to perform phase II studies. We decided to test a new reduction-remission regimen as primary treatment in the same patient population participating in a previous MDS study [17]. We elected to incorporate gemtuzumab ozogamicin (GO) into regular remission-induction regimen. GO consists of a humanized anti-CD33 monoclonal antibody linked to calicheamicin, a potent antitumor antibiotic [18]. GO binds to CD33, an antigen expressed on the surface of >90 % of AML blast cells. Binding of GO is followed by internalization and toxin release intracellularly leading to DNA damage and cell death [19]. In studies of older patients with AML in first relapse, tolerable toxicity and a response rate of 30 % was reported following two infusions of GO 9 mg/m^2^, although full platelet recovery did not occur in roughly half of responders [20]. These results led to the drug regulatory approval in the United States for use in older patients in first relapse for whom standard therapy was unsuitable, setting the stage for its evaluation in patients with newly diagnosed high-risk MDS. Our group developed a prospective randomized trial combining two infusions of GO 6 mg/m^2^ followed by standard remission-induction chemotherapy in fit older patients [21]. This combination provided no benefit compared to the control arm and appeared too (hemato)toxic in patients older than 70 years. Patients with secondary AML younger than 70 years might be an exception [22]. Based on this experience, we selected the GO dosage of 5 mg/m^2^ as a single infusion on day 7 of the chemotherapy regimen [21, 23].

## Patients and methods

### Patients and eligibility

Thirty-one patients were registered in this study between January 2003 and March 2006. One patient was ineligible. Therefore, a total number of 30 patients were evaluable. The patients had to meet the following disease criteria: (1) high-risk MDS, defined as refractory anemia with excess of blasts in transformation (RAEBt), RAEB > 10 % BM blasts, other forms of MDS with multiple (≥3) chromosomal abnormalities or chromosome seven abnormalities and/or severe cytopenias defined as follows: neutrophil count <0.5 × 10^9^/l and/or platelet count <20 × 10^9^/l; (2) chronic myelomonocytic leukemia (CMMoL) with >5 % BM blasts or with >16 × 10^9^/l neutrophils or with 2.6 × 10^9^/l monocytes in the blood; (3) secondary AML after overt MDS of more than 6 months duration. In addition, the following criteria were required: age 16 to 70 years and adequate renal and liver function, defined as 1.5 × upper limit of normal (ULN). Patients who had already received chemotherapy and/or radiotherapy were not eligible. All participants gave their informed consent. The study was registered in clinicaltrials.gov (NCT00077116).

### Study design

The cytarabine in combination with gemtuzumab ozogamicin (IAGO) study was a phase II study carried out by the European Organization for Research and Treatment of Cancer (EORTC) Leukemia group and the Gruppo Italiano Malattie Ematologiche dell Ádulto (GIMEMA).

The primary objective of this trial was to assess the feasibility, toxicity profile, and antileukemic/anti-MDS activity of GO in combination with a standard chemotherapy regimen consisting of idarubicin and cytarabine in previously untreated patients with high-risk MDS or sAML developing after a preceding period with MDS during 6 months.

Secondary objectives were to monitor hepatotoxicity, in particular veno-occlusive disease (VOD), to determine the severity of pancytopenia and duration of recovery in patients who reached complete remission or CR with incomplete hematopoietic recovery (CRi).

Patients who met eligibility criteria had to be prospectively registered at the EORTC Headquarters in Brussels, Belgium.

The remission-induction course consisted of a continuous infusion of cytarabine 100 mg/m^2^/day for 10 days in combination with idarubicin 12 mg/m^2^/day on days 1, 3, and 5 as 5-min infusions and GO on day 7 as a 1-h infusion at the dose level of 5 mg/m^2^ (IAGO).

Response assessment was planned around day 31 after the start of the induction course. The revised recommendations of the International Working Group for Diagnosis, Standardization of Response Criteria, Treatment Outcomes, and Reporting Standards for Therapeutic Trials in MDS [24] were used. A CR required normalization of the marrow blasts (less than 5 %) and recovery of normal hematopoiesis with a neutrophil count of 1 × 10^9^/l or more and a platelet count of 100 × 10^9^/l or more in addition to disappearance of all clinical, laboratory, or radiologic evidence of disease. CRi had criteria similar to CR but with neutrophils between 0.5 × 10^9^ and 1 × 10^9^/l and/or platelet counts between 50 × 10^9^ and 100 × 10^9^/L. Partial remission (PR) required blood recovery as for CR but with both a decrease in marrow blasts of at least 50 % and not more than 25 % abnormal cells in the marrow.

In case PR was achieved after the first course, a similar second remission-induction course was given. In case of CR or CRi after one or two induction courses, a single consolidation course was recommended consisting of intermediate-dose cytarabine (500 mg/m^2^ every 12 h in a 2-h infusion, on days 1–6) and idarubicin (10 mg/m^2^/day as 5-min infusion, on days 4, 5, 6) to be followed by either an allogeneic HSCT or an autologous HSCT [17].

Reasons to stop treatment in the protocol: normal completion of the protocol after one or two courses IAGO, ineligibility, non-compliance of the patient, excessive toxicity, death, and loss to follow-up (LFU).

### Endpoints

The main endpoints were the best complete response (CR/CRi) rate after one or two courses of IAGO and severe toxicity rate observed during or after the completion of IAGO. The secondary endpoint(s) were the following: survival and progression-free survival (PFS) from start of treatment. Overall survival (OS) was calculated, from the date of start of treatment until date of death (whatever the cause). Patients still alive were censored at the moment of last visit/contact.

PFS was calculated from the start of the first course of IAGO until first date of relapse in patients who reached CR/CRi or progression or until death (whatever the cause and whichever occurred first). Patients still alive, in first CR/CRi (i.e., without relapse), have been censored at the moment of last visit/contact.

Adverse events for each treatment course were recorded according to the National Cancer Institute’s Common Terminology Criteria for Adverse Events (version 2.0).

### Statistical design and methods

A Bryant-Day one-step design was used, including response (CR/CRi) and excessive toxicity as the primary endpoint. The following design parameters were considered:

- P0 is the largest CR/CRi probability which, if true, implies that the therapeutic activity does not warrant further investigation of the regimen (IAGO). In the present trial, P0 has been taken as 50 %.
- P1 is the lowest CR/CRi probability which, if true, implies that the therapeutic activity does warrant further investigation of the regimen provided acceptable severe acute toxicity occurrence. In the present trial, P1 has been taken as 75 %.
- (1-T0) is the smallest severe toxicity occurrence probability which, if true, implies that the therapeutic toxicity is unacceptable and the regimen does not warrant further investigation. In the present trial, (1-T0) was taken as 50 %.
- (1-T1) is the largest severe toxicity occurrence probability which, if true, implies that the therapeutic toxicity is acceptable and the regimen does warrant further investigation provided acceptable response rate. In the present trial, (1-T1) was taken as 20 %.
- Beta error: the accepted probability of rejecting from further trials a regimen with a true CR/CRi rate at least equal to P1 and a true toxicity rate equal to or lower than (1-T1). In the present trial, beta was taken as 0.10.
- Alpha error: the accepted probability of recommending for further investigation a regimen with a true CR/CRi rate equal to or lower than P0. It is also the accepted probability of recommending for further trials a regimen with a true severe toxicity rate equal to or higher than (1-T0). In the present trial, the two alphas were taken as 0.10.

Based on these parameters, a total of 28 patients had to be assessed for overall response and toxicity, and the following decision rule had to be applied:

- If ≤17 (17/28 = 60.7 %), CR/CRis were observed, or if ≥11 (11/28 = 39.3 %), patients had severe toxicities, the conclusion that IAGO is not enough active or is too toxic, and should not be further investigated; otherwise, the conclusion will be that IAGO is active and feasible, and should be further investigated in this patient population.

A total of 31 patients has been finally been entered, in order to cope with the exclusion of ineligible patients or of those who did not start IAGO course. Among them, one patient was considered to be ineligible by the study coordinator because of incorrect diagnosis.

The time to event distributions (PFS and OS) were estimated using the Kaplan-Meier technique, and the standard errors (SE) of the estimates were obtained via the Greenwood formula. SAS 9.3 software (SAS Institute, Cary, NC) was used for the statistical analyses.

The CRIANT score was used to analyze the outcome according to adapted prognostic criteria [2]. The score was created based on the weight expressed in points of five criteria: cytogenetics (good, 0; intermediate, 20; poor, 40; unknown, 20), WBC × 10^9^/l (<25, 0; ≥25, 20), age in years (≤45, 0; 45–55, 20; >55, 22), antecedent hematological disorder in months (≤6, 0; >6, 13), and number of cytopenias (0–2, 0; 3, 15). The CRIANT score distinguished three risk groups: low risk, <20 points; intermediate risk, 20–49 points; and high risk, ≥50 points.

## Results

**Fig. 1:**
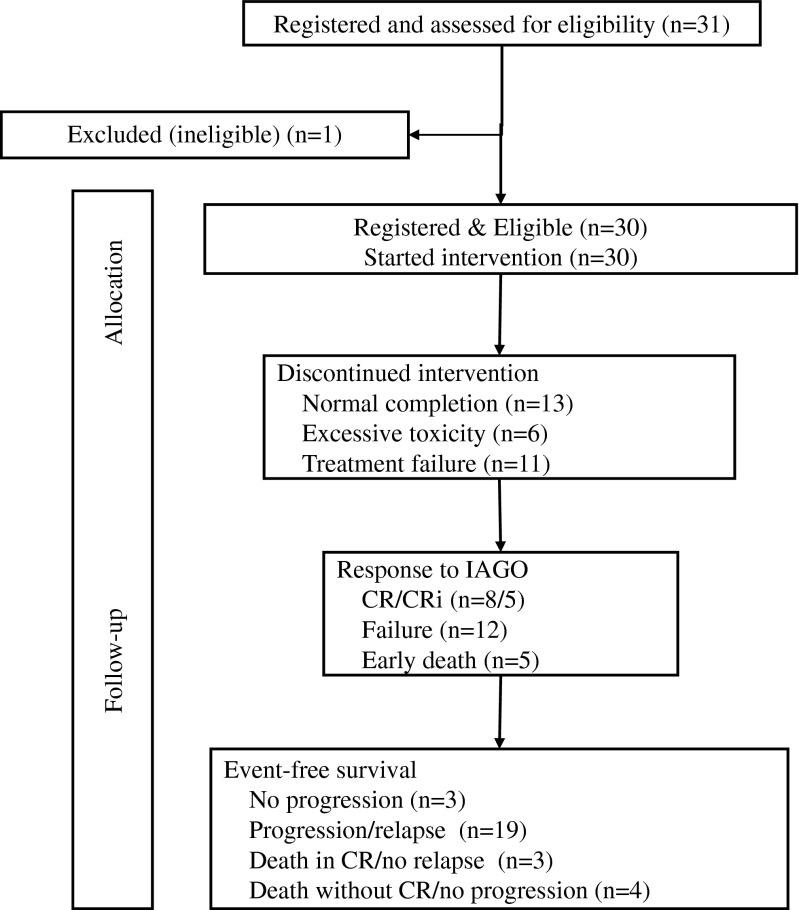
Treatment plan and flow diagram

Patient characteristics are shown in Table 1. The median age was 58 years with a range from 21 to 66 years. Fourteen patients had progressed to RAEBt [5] or secondary AML [9]. All 30 eligible patients started the planned treatment (Fig. 1). All patients received the full dosage of idarubicin, two patients received a slightly modified dosage of cytarabine, and one patient a modified dosage of GO. Three patients received a second course of IAGO after a PR to the first course.

**Table 1:** Patient characteristics and survival rates at 1 and 2 years

Patient characteristics	All patients (%)	Survival (SE)	
		At 1 year	At 2 years
All patients (M/F)	30 (21/9)	53 (9)	27 (8)
Age <55 years		44 (17)	33 (16)
Age ≥55 years		57 (11)	24 (9)
Performance status (WHO, 0–4)			
0	17	–	–
1/2	3	–	–
MDS/sAML classification	21	48 (11)	24 (9)
RAEB-1	1	–	–
RAEB-2	12	–	–
CMML <5 % marrow blasts	1	–	–
CMML ≥5 % marrow blasts	2	–	–
RAEBt/sAML	14	64 (15)	–
Hemoglobin (g/dl) <10	25	–	–
Hemoglobin (g/dl) ≥10	5	–	–
All nucleated cells <1.8 × 10^9^/l	17	–	–
All nucleated cells ≥1.8 × 10^9^/l	3	–	–
Platelets <100 × 10^9^/l	21	–	–
Platelets ≥100 × 10^9^/l	9	–	–
Cytogenetics (IPSS)			
Good	10	90 (9)	40 (15)
Intermediate	9	56 (17)	33 (16)
Poor	7	0	0
ND/failure	4	50 (25)	25 (22)
IPSS			
Intermediate-1	2	50 (35)	50 (35)
Intermediate-2	6	67 (19)	33 (19)
Poor	13	39 (13)	15 (10)
sAML	9	67 (16)	33 (16)
Interval from diagnosis to start of treatment			
≤2 months	15	–	–
>2 months (range 2–53)	15	–	–
CRIANT score (points)			
0–20	2 (7)	54 (14)	23 (12)
20−<50	13 (43)	47 (13)	20 (10)
≥50	15 (50)	47 (13)	33 (16)

*SE* standard error, *RAEB* refractory anemia with excess of blasts, *CMML* chronic myelomonocytic leukemia, *RAEBt* RAEB in transformation, *sAML* secondary acute myeloid leukemia, *IPSS* international prognostic scoring system, *CRIANT Score* for details, see “Methods” section

### Side effects

Side effects occurred in 25 patients during and after the first course, including grade 3 to 4 toxicities in nine patients (see Table 2). Besides fatigue, the most prominent toxicity concerned liver toxicity, as shown by elevated bilirubin levels in 16 patients, including grade 3 to 4 in five patients. One case of clinically relevant VOD has been diagnosed during the first course of IAGO. This patient recovered completely, and he could complete his treatment (see Table 3). Two out of the three patients who received the second course developed severe liver toxicity as shown by grade 3–4 elevated bilirubin levels, but these patients did not develop VOD. Five (17 %) patients died within 40 days after start of IAGO-1 due to treatment related toxicities; for details, see Table 3.

**Table 2:** Overview of toxicities during IAGO-1 course

Type of toxicity	All grades	Grades 3–4
All	30	
Allergy	3	–
Hypertension		–
Cardiac	5	
Fatigue	16	7
Skin rash	5	2
Anorexia	15	4
Stomatitis/mucositis	13	4
Hemorrhage	4	2
Veno-occlusive disease	Not applicable	1
Febrile neutropenia	9	9
Elevated bilirubin	16	5
Elevated creatinine	5	–
Elevated AST	18	2
Elevated ALT	16	2

**Table 3:** Overview of outcome of nine patients with severe toxicities

Pt nr	Duration survival (days)	Main complication/reason off protocol
2	23	Febrile neutropenia leading to diffuse intravascular coagulation and to liver and renal failure; VOD not likely
6	32	Cardiac arrest
9	11	Pulmonary infiltrates; died from pulmonary failure due capillary leakage
13	1129	VOD; normal completion of protocol
20	1254+	Bilateral interstitial pneumonitis + normal completion
24	37	Lung infection, pulmonary insufficiency, and multi-organ failure
25	1035+	Iatrogenic hematothorax + normal completion
26	103	Grade 3 bilirubin elevation and liver lesions; grade 3 infection
29	36	Cerebral hemorrhage day 35; persisting thrombocytopenia

### Responses and duration of hypoplasia

Thirteen (43, 90 % CI (28, 60 %)) patients achieved a major response, including eight patients with CR and five patients with CRi (Fig. 1). Among the latter group, one patient achieved PR after one course and entered CRi after two courses of the IAGO schedule. CR or CRi only occurred in patients with good and intermediate cytogenetic risk score according to IPSS (six out of 10 and five out nine, respectively), while none of the seven patients with poor-risk cytogenetic characteristics achieved CR in this study. The hematopoietic recovery of patients who achieved CR or CRi was clearly delayed as shown by the median recovery of neutrophils to 0.5 × 10^9^/l of 30 days and of the platelets to >50 × 10^9^/l of 29 days. For further details, see Table 4. Thirteen patients (43 %) had a normal completion of the protocol consisting of CR or CRi (Fig. 1). The other patients went off protocol due to toxicity in six patients or due to treatment failure in 11 patients (37 %) (Fig. 1).

**Table 4:** Hematopoietic recovery after first course of IAGO in 13 patients with CR or CRi

Recovery from start of IAGO	Medium number of days (95 % confidence intervals)
PMN >0.5 × 10^9^/l	30 (28 to 32)
PMN >1.5 × 10^9^/l	35 (31 to 43)
Platelets >50 × 10^9^/l	29 (28 to NR)
Platelets >100 × 10^9^/l	42 (28 to NR)

*PMN* polymorphic nucleated cells, *NR* not reached

### Post-remission therapy

All 13 patients with CR/CRi received consolidation therapy which was followed by allogeneic HSCT in five patients and autologous HSCT in three patients. Another patient who failed to respond to the induction also received an allogeneic transplantation. Five patients are alive without evidence of disease, including one patient (nr 20) in second CR after alloSCT; for details, see Table 5. Three patients have died in CR due to complications after HSCT: two patients after alloHSCT and one patient after autoHSCT.

**Table 5:** Post-remission therapy and outcome

Pt nr	Response	Type of SCT	SCT^a^	Relapse^a^	Survival^a^: cause of death^b^
4	CR	Auto	120	209	269
10	CR	No		336	490
11	CR	Auto	194	No	203: infection
13	CRi	No		414	1129
15	CRi	No		No	1463^c^: CR
16	CR	Allo	173	No	1420^c^: CR
17	CR	Auto	127	132	160
19	CR	Allo	205	No	441: GVHD
20	CRi	Allo	576	471	1254^c^: second CR
22	CR	No		305	405
23	CR	Allo	160	No	168: NRM
25	CRi	No		No	1035^c^: CR
28	CRi	Allo	103	No	1042^c^: CR
31	Failure	Allo	161	Progression	827^c^: active disease

*CR* complete remission, *CRi* CR with incomplete recovery of platelets and leukocytes

^a^Number of days after starting treatment

^b^Active disease, if not mentioned specifically

^c^Patient still alive at last follow-up

### Outcome and prognostic factors

**Fig. 2:**
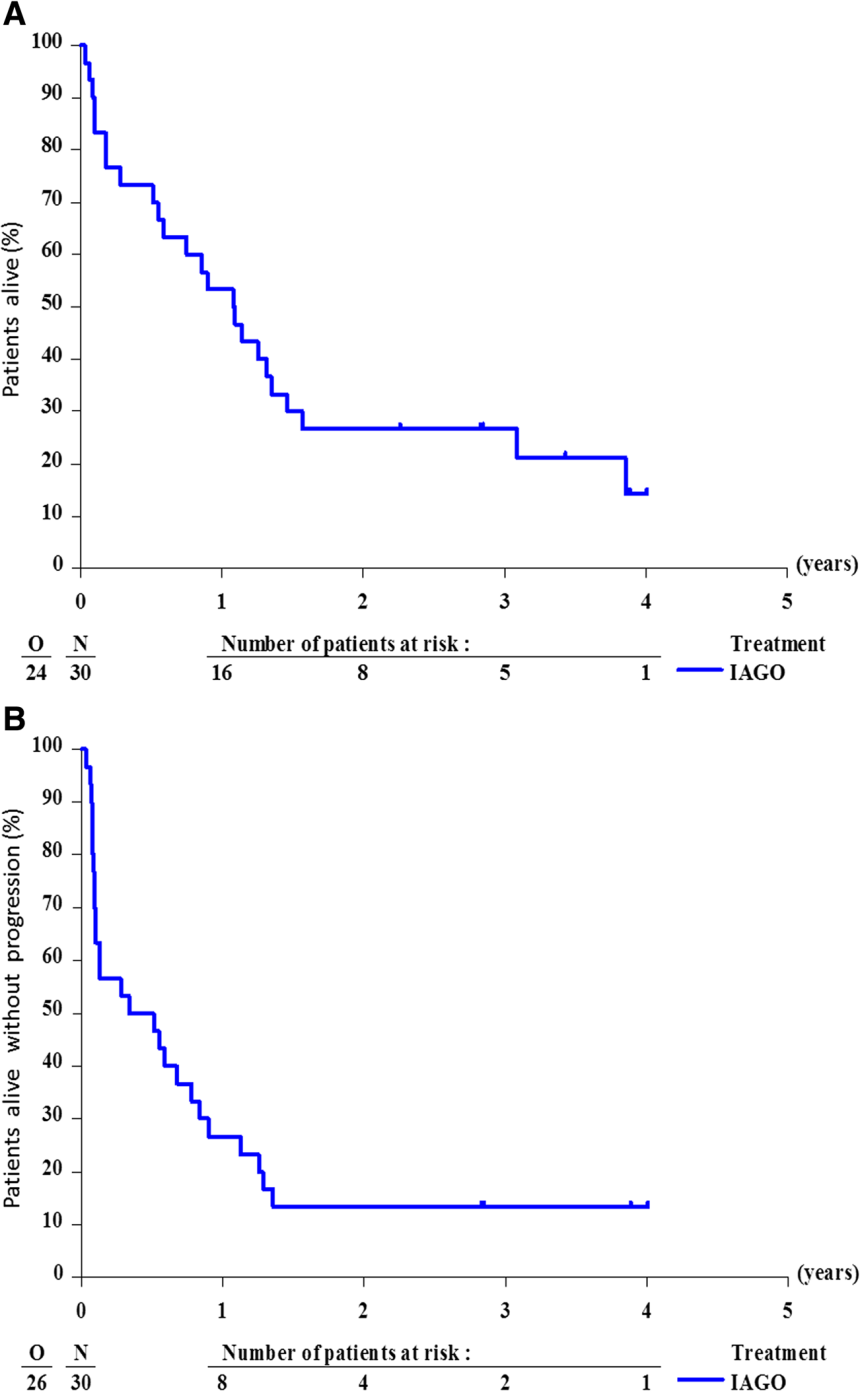
**a** Overall survival after treatment with IAGO and post-remission therapy. **b** Progression-free survival after treatment with IAGO and post-remission therapy

At the time of final evaluation, four patients (13 %) were still alive without signs of progression and two additional patients were alive either with active disease or in second CR (Table 5). Seventeen patients have died with active disease and seven patients due to toxicity, including three patients in CR. The median follow-up was 3.4 years. The median survival was 1.09 years (95 % CI 0.75 to 1.57). The survival rate at 1 year from start of treatment was 53 % (SE 9 %) and at 2 years was 27 % (SE 8 %) (Table 1, Fig. 2a).

The median PFS was 5.1 months (95 % CI 1.6 to 10.8), and the PFS rate at 1 year was 27 % with SE of 8 % (Fig. 2b).

**Fig. 3:**
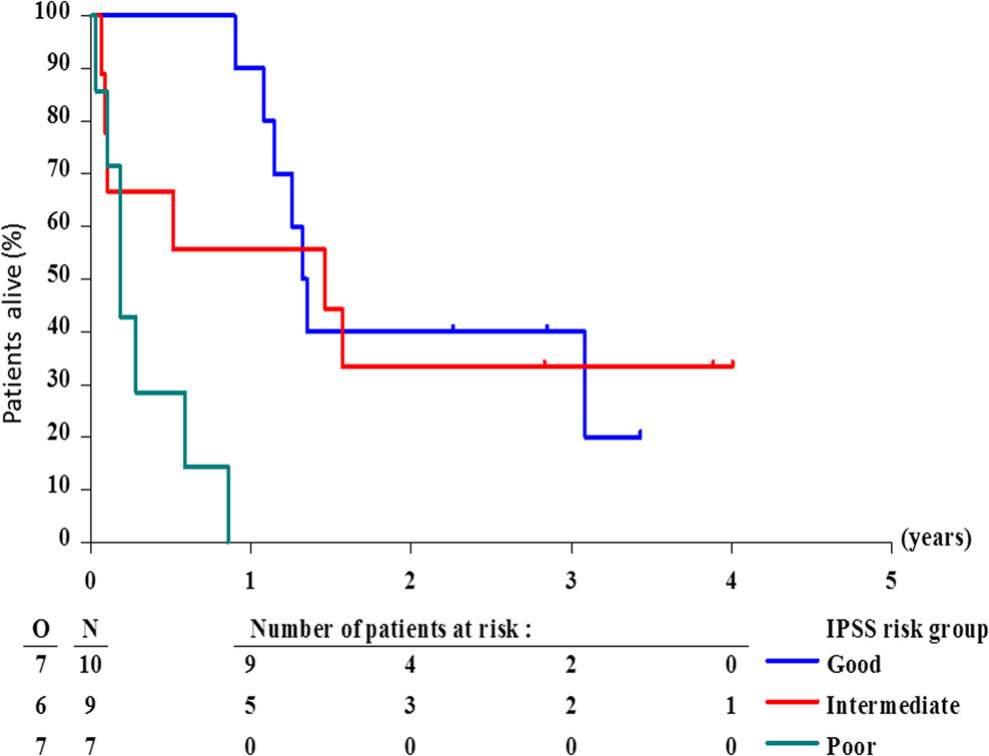
Overall survival in cytogenetic subgroups according to IPSS

Age (<55 versus ≥55 years) had no impact on survival (Table 1). None of the seven patients with IPSS poor-risk cytogenetic characteristics was alive at 1 year after start of IAGO, while the 1-year survival in patients with good or intermediate risk cytogenetics was 90.0 % (SE 9 %) and 56 % (SE 17 %), respectively; for details and 2-year survival rates, see Table 1 and Fig. 3. The percentage of marrow blasts, subdivided according to less than 20 % or 20 % and more did not influence survival, with a 1-year survival rate of 47 % (SE 11) and 64 % (SE 15 %), respectively. The prognostic importance of IPSS regarding survival was weak, probably due to the important weight allocated to the percentage of marrow blasts and/or due to the limited number of patients in each IPSS subgroup (Table 1). Fifty percent of the patients were classified as poor-risk according to the recently developed CRIANT risk score [2] and only two patients as good-risk patients. The survival of the poor-risk and intermediate risk patients was similar (Table 1).

## Discussion

In view of the observed CR/CRi rate of 43 % (13 out of 30 patients) with a 90 % CI of (27.9, 59.8 %), which did not contain the targeted CR/CRi rate of 75 %, the Bryant-Day one-step (negative) decision rule of this study was met. This CR rate is lower than the generally observed remission rates after various intensive chemotherapy regimens [12, 17, 20, 25, 26]. This indicates that the IAGO schedule was not sufficiently active to warrant further exploration in this category of patients.

The major cause of treatment failure in this study was insufficient efficacy of the IAGO schedule. Similar studies, exploring new schedules in high-risk MDS patients, have resulted in higher CR rates, including the 71 % CR rate in the FLAG schedule (fludarabine, high dose cytarabine, and G-CSF) [26]. Several randomized studies of GO combined with intensive chemotherapy have been performed in patients with elderly primary AML. A meta-analysis of five prospective studies (in total 3325 patients) [27] showed that addition of GO did not increase the proportion of patients entering CR with an odds ratio (OR) of 0.91 and a 95 % confidence interval (CI) of 0.77–1.07 (*p* = 0.3). However, the addition of GO significantly improved survival (OR 0.90; 95 % CI 0.82–0.98, *p* = 0.03), although the 5-year OS difference was only around 3 %: 35.5 % (GO arm) versus 32.2 % (control group). In addition, patients with adverse cytogenetic characteristics did not benefit from the addition of GO in contrast to patients with favorable or intermediate cytogenetic features [27]. The great majority of patients in this meta-analysis were patients with de novo AML. Only two studies [28, 29] included patients with secondary AML and only one study included high-risk MDS patients [29].

Toxicity was an important contributing factor to the general outcome in this study, since six out of the 17 patients went off protocol due to toxicity. Toxicity was fatal within 40 days after starting treatment in five patients (16.7 %). Overall, the observed grade 3–4 toxicity was 33.3 %, and its 90 % CI (16.6, 46.5 %) did not cover the inacceptable toxicity rate of 50 %. In an earlier study of our group [30], we treated a younger age group (median age of 47 years) of 194 patients with the same remission-induction regimen (idarubicin, cytarabine, and etoposide (ICE)) with the exception of etoposide which has been replaced by GO in the present study. The CR rate in that study was 54 % and 29 patients (16 %) died during the remission-induction phase, which is comparable to the 17 % observed in the current study [30]. The CR rate was 58 % in a more recent study [17] utilizing the same schedule (ICE) as the previous study [30]. The median patient age of the patients in this study was lower (52 versus 58 years), but the percentage of patients with poor-risk cytogenetics was higher in the previous study: 31 versus 23 % in the IAGO study [17]. None of the seven patients with poor-risk cytogenetic characteristics entered CR/CRi, and none of these patients was alive at 1 year after starting treatment. In the previous study, three risk groups were distinguished: a good-risk group with a score <20, an intermediate risk group with a score 20–<50, and a poor-risk group with a score ≥50. The 5-year estimated survival rates were 69 % (SE = 10.2 %), 37 % (SE = 5.6 %), and 5 % (SE = 2.1 %) for the three groups, respectively [2]. Poor-risk cytogenetic characteristics had a pronounced weight in this scoring system. Only two patients in this study were classified as good risk and 15 patients as poor-risk. The survival of the intermediate and poor-risk patients was not significantly different (*p* = 0.29), but the numbers are small (Table 1).

GO has been administered as a single dose of 5 mg/m^2^ on day 7 of the chemotherapy schedule in the current study. It is possible that a more fractioned schedule with a higher total dose of GO might be a more effective strategy which may take advantage of CD33-re-expression that occurs after initial exposure to the GO [31]. The French Alfa-group utilized a GO schedule of 3 mg/m^2^/day on days 1, 4, and 7 during induction chemotherapy in patients aged 50–70 years with untreated primary AML. Complete response was 81 % and event-free survival was 40.8 % compared to 17.1 % in the control group (*p* value = 0.003) [32]. This benefit was also apparent in patients with unfavorable cytogenetic characteristics, but the impact of complex karyotype has not been analyzed separately [32]. In addition, early mortality seems to be reduced if a dose of 3 g/m^2^ is used either as a single dose or in a fractionated schedule [27].

## Conclusions

The observed CR/CRi’s rate of 43 % led to the conclusion that IAGO is not sufficiently active to warrant further exploration in high-risk MDS and secondary AML evolved from MDS. In addition, patients with poor cytogenetic features had a very poor outcome caused by a complete absence CR and CRi rate in this phase II study. Alternative schedules with more fractionated schedules of GO may result in better outcome in this population of high-risk MDS or AML evolved from MDS.
